# Long-Term Outcomes Associated With β-Lactam Allergies

**DOI:** 10.1001/jamanetworkopen.2024.12313

**Published:** 2024-05-17

**Authors:** Matthew P. Gray, John A. Kellum, Levent Kirisci, Richard D. Boyce, Sandra L. Kane-Gill

**Affiliations:** 1 Department of Pharmacy and Therapeutics, University of Pittsburgh School of Pharmacy, Pittsburgh, Pennsylvania; 2 Department of Critical Care Medicine, University of Pittsburgh School of Medicine, Pittsburgh, Pennsylvania; 3 Department of Biomedical Informatics, University of Pittsburgh School of Medicine, Pittsburgh, Pennsylvania

## Abstract

**Importance:**

β-lactam (BL) allergies are the most common drug allergy worldwide, but most are reported in error. BL allergies are also well-established risk factors for adverse drug events and antibiotic-resistant infections during inpatient health care encounters, but the understanding of the long-term outcomes of patients with BL allergies remains limited.

**Objective:**

To evaluate the long-term clinical outcomes of patients with BL allergies.

**Design, Setting, and Participants:**

This longitudinal retrospective cohort study was conducted at a single regional health care system in western Pennsylvania. Electronic health records were analyzed for patients who had an index encounter with a diagnosis of sepsis, pneumonia, or urinary tract infection between 2007 and 2008. Patients were followed-up until death or the end of 2018. Data analysis was performed from January 2022 to January 2024.

**Exposure:**

The presence of any BL class antibiotic in the allergy section of a patient’s electronic health record, evaluated at the earliest occurring observed health care encounter.

**Main Outcomes and Measures:**

The primary outcome was all-cause mortality, derived from the Social Security Death Index. Secondary outcomes were defined using laboratory and microbiology results and included infection with methicillin-resistant *Staphylococcus aureus* (MRSA), *Clostridium difficile*, or vancomycin-resistant *Enterococcus* (VRE) and severity and occurrence of acute kidney injury (AKI). Generalized estimating equations with a patient-level panel variable and time exposure offset were used to evaluate the odds of occurrence of each outcome between allergy groups.

**Results:**

A total of 20 092 patients (mean [SD] age, 62.9 [19.7] years; 12 231 female [60.9%]), of whom 4211 (21.0%) had BL documented allergy and 15 881 (79.0%) did not, met the inclusion criteria. A total of 3513 patients (17.5%) were Black, 15 358 (76.4%) were White, and 1221 (6.0%) were another race. Using generalized estimating equations, documented BL allergies were not significantly associated with the odds of mortality (odds ratio [OR], 1.02; 95% CI, 0.96-1.09). BL allergies were associated with increased odds of MRSA infection (OR, 1.44; 95% CI, 1.36-1.53), VRE infection (OR, 1.18; 95% CI, 1.05-1.32), and the pooled rate of the 3 evaluated antibiotic-resistant infections (OR, 1.33; 95% CI, 1.30-1.36) but were not associated with *C difficile* infection (OR, 1.04; 95% CI, 0.94-1.16), stage 2 and 3 AKI (OR, 1.02; 95% CI, 0.96-1.10), or stage 3 AKI (OR, 1.06; 95% CI, 0.98-1.14).

**Conclusions and Relevance:**

Documented BL allergies were not associated with the long-term odds of mortality but were associated with antibiotic-resistant infections. Health systems should emphasize accurate allergy documentation and reduce unnecessary BL avoidance.

## Key Points

**Question:**

What are the long-term clinical outcomes of patients with β-lactam allergies?

**Findings:**

In this cohort study following 20 092 adult patients for up to 12 years, a generalized estimating equation analysis model found that the presence of a documented β-lactam allergy was not associated with a statistically significant increase in the odds of all-cause mortality but was associated with an increase in antibiotic-resistant infections.

**Meaning:**

These findings suggest that documented β-lactam allergies are associated with substantial long-term clinical detriment, and health systems should prioritize initiatives to maximize the use of first-line antimicrobials and reduce unnecessary β-lactam avoidance.

## Teaser

This cohort study examines long-term clinical outcomes, including mortality and antibiotic-resistant infection with methicillin-resistant *Staphylococcus aureus*, *Clostridium difficile*, or vancomycin-resistant *Enterococcus*, for adults with β-lactam allergies living in western Pennsylvania.

## Introduction

Allergies to β-lactam (BL) class antibiotics are the most commonly reported drug allergy in the US, with 5% to 13% of the population reporting an allergy to at least 1 BL-class antibiotic.^1,2,3,4^ Patients with documented BL allergies incur increased rates of readmission, antibiotic-resistant infections, adverse events, and longer lengths of stay (LOSs) compared with patients without documented BL allergies.^3,5,6,7,8^ As a result of the harm associated with inpatient encounters for patients with documented BL allergies, there has been a large increase in initiatives that emphasize the delabeling of erroneous BL allergies, which are reported in error for more than 90% of the patients with documented BL allergies.^9,10,11^ However, the long-term clinical impact of BL allergies remain poorly understood.^12^

To our knowledge, there has been only one previous set of cohort studies^13,14^ that evaluated the long-term outcomes associated with the presence of a documented BL allergy, and those studies found that BL allergies were associated with a 14% increase in the hazards of mortality, a 69% increase in the hazards of infection with methicillin-resistant S*taphylococcus aureus* (MRSA), and a 26% increase in the risk of infection with *Clostridium difficile*. The poor documentation of BL allergies, which is often based on outdated and unreliable historical information, may be leading to the use of onetime stopgaps for immediate management, while allowing the allergy to remain present in the patient’s electronic health record (EHR) and lead to long-term harm through altered prescribing patterns.^15,16,17^ BL allergies, and drug allergies in general, should be considered a long-term risk factor because more than 80% of all documented drug allergies are never delabeled.^18^ Because BL antimicrobials are so frequently used, and drug allergies are rarely removed, more long-term evaluations of patients with BL allergies are required to understand the full scale of harm they may be causing on the population.

We sought to expand the understanding of the long-term clinical outcomes associated with patients who have a documented BL allergy. The current understanding of the long-term clinical outcomes of BL allergies is limited to one set of UK-based cohort studies,^13,14^ and given the scope and impact of BL allergies globally, further studies examining additional outcomes and using different populations are required. Our study aimed to expand upon these previous studies by using a cohort of regionally based health care encounters from the US and including secondary clinical outcomes of acute kidney injury (AKI) and additional antibiotic-resistant infections. Furthermore, our study identified patients for inclusion through a 2-year span of patients with targeted infectious diseases of pneumonia, urinary tract infection (UTI), and sepsis, which may increase the likelihood that included patients had their care affected by the presence of a BL allergy.

## Methods

### Study Design

We performed a retrospective cohort analysis using EHR data from 2007 to 2018. The University of Pittsburgh institutional review board reviewed the study protocol, and it was assigned exempt status. Informed consent was waived because the data were deidentified, in accordance with 45 CFR §46. This study was designed and conducted in accordance with the Reporting of Studies Conducted using Observational Routinely-Collected Data (RECORD) reporting guidelines for studies using health data,^19^ which is an extension of the Strengthening the Reporting of Observational Studies in Epidemiology (STROBE) reporting guidelines.^20^

### Data Cohort

Data were collected for a cohort of deidentified patient encounters occurring between 2007 and 2018 that was derived from the Medical Archival Retrieval System, an electronic medical data repository for patients from University of Pittsburgh Medical Center, a health system based in Western Pennsylvania.^21,22^ During the study, included hospitals were transitioning to a new EHR system, and a total of 16 hospitals, which ranged in size from 40 to 900 beds, were included on an ongoing basis as their data became available. Patients were identified for inclusion through a hospital-based health care encounter between 2007 and 2008 with a diagnosis of sepsis, pneumonia, or UTI. Sepsis was defined using a previously established method requiring an *International Classification of Diseases, Ninth Revision (ICD-9)* code indicating infection and an *ICD-9* code indicating organ damage (excluding mechanical ventilation codes).^23^ Pneumonia and UTI were similarly identified using previously published methods for identification of these diagnoses using *ICD-9* codes.^24,25^ A full* ICD-9* code list is shown in eTable 1 in Supplement 1. The cohort was limited to patients who were aged at least 18 years and had allergy information available for the index encounter. Included patients were followed-up until the earliest occurrence of 2 events: the end of 2018 or death.

### Variables

BL allergy status was defined as the presence of any penicillin, cephalosporin, carbapenem, or aztreonam as an allergy in the patient’s allergy list. Allergy lists were derived from pharmacy discharge summaries and were recorded as free text. An online medication repository was used to assist in creating a full list of applicable products to search for allergies.^26^ Over the course of follow-up, 955 patients had their BL allergy status vary from the first observed encounter (eg, a BL-allergic patient had their documented allergy removed, or vice versa). In these situations, each patient’s initial allergy status was carried forward for the full follow-up period.

Independent variables included age, sex, race, baseline serum creatinine (SCr) level, Van Walraven comorbidity score and Elixhauser Comorbidity Index score, the number of health care encounters per patient, an indicator for intensive care unit (ICU) admission, dialysis utilization during each encounter, and an indicator for the individual hospital at which the health care encounter occurred.^27,28,29^ Hospital locations were referenced only as numeric indicators to protect patient identifiability. There were instances where some secondary outcomes were not observed at individual hospitals, meaning that encounters occurring at these hospitals could not be included for analysis when the outcome was not observed. Race was identified from the EHR and was condensed into 3 categories: Black, White, or other, which includes Aleutian-Indian, Asian, Chinese, Eskimo-Indian, Filipino, Hawaiian, Hispanic, Japanese, Middle Eastern, Oriental, Pacific Islander, multiracial, unknown, and other or declined. Data on race are included in this study because race can be a risk factor for AKI.^31^ Baseline SCr was calculated by first determining whether a patient had a stable or unstable admission SCr, with an unstable SCr defined as a change in SCr greater than 0.3 mg/dL (to convert to micromoles per liter, multiply by 88.4) within 48 hours following the first recorded SCr. Those with stable admission SCr had their first admission SCr used as the baseline, and those with unstable SCr had a baseline SCr calculated using the Modification of Diet in Renal Disease equation.^30^

### Outcomes

The primary outcome was all-cause mortality, defined using data from the Social Security Death Index. Secondary outcomes included the occurrence of AKI, grouped as stage 2 and 3 or stage 3 AKI defined by the Kidney Disease Improving Global Outcomes 2012 guidelines.^31^ Only follow-up encounters with at least one available SCr measurement were considered for analysis for AKI. Additional secondary outcomes included occurrence of infection with MRSA, *C difficile*, or vancomycin-resistant *Enterococcus* (VRE), that were defined using corresponding system-level microbiology codes, and the pooled occurrence of any of these 3 antibiotic-resistant infections.

### Statistical Analysis

The analysis was performed between January 2022 and January 2024. Statistical analysis was conducted using Stata statistical software version 18.0 (StataCorp).^32^ Statistical significance was defined a priori as *P* < .05 using 2-sided statistical hypothesis testing. We evaluated baseline demographic and clinical characteristics at the earliest occurring encounter for each patient. These were compared for significance using 2-sample *t* tests for continuous variables and χ^2^ tests for binary variables. Missingness for age (<1%) and baseline SCr (22%) at baseline were imputed using multiple imputation by chained equation with predictive mean matching.^33,34^ We used multivariable generalized estimating equations to model the outcomes.^35^ A panel variable was included at the patient level, and all models used logit link functions and independent correlation structures. The time in days that each patient was exposed to each state was included as an offset variable. We analyzed patient data from the time of their index health care encounter to the first occurring instance of death or the end of the study period.

Outcomes were evaluated independently at each encounter, meaning that outcomes except death could recur within the same patient at different encounters. Bootstrapping (100 iterations) was used to calculate alternative 95% CIs. A sensitivity analysis was performed to compare the effect of analyzing BL allergy status as a dynamic variable, allowing patient allergy status to vary over time. For this sensitivity analysis, each patient’s BL allergy status was evaluated independently at each health care encounter, allowing it to vary over time, and the primary model was then repeated using this dynamic allergy variable. An additional sensitivity analysis was conducted in only the population for whom their allergy status changed over the follow-up period, as well as patients whose allergy status never changed. Because of the large amount of missingness in baseline SCr data that required imputation (22%), a sensitivity analysis was conducted to evaluate the results while not including baseline SCr as an independent variable. Finally, LOS was considered as a confounder and included as a covariate in a sensitivity analysis. We were unable to include LOS in the primary analysis owing to emergency department and same-day-surgery encounters not having a calculable LOS, and for this sensitivity analysis, we constrained LOS to have a value of 1 day if it was not able to be calculated.

## Results

### Cohort Characteristics

A total of 20 092 patients (mean [SD] age, 62.9 [19.7] years; 12 231 female [60.9%]) were identified who met the inclusion criteria, including 4211 (21.0%) patients who had a documented BL allergy upon their first observed encounter and 15 881 (79.0%) who did not. A total of 3513 patients (17.5%) were Black, 15 358 (76.4%) were White, and 1221 (6.0%) were another race. Patients with a documented BL allergy were more likely to be female (2911 patients [69.1%] vs 9320 patients [58.7%]), older (mean [SD] age, 64.2 [19.9] years vs 62.6 [19.0] years), and White race (3332 patients [79.1%] vs 12 026 patients [75.7%]) than those without a BL allergy. Baseline clinical characteristics, including baseline SCr level (median [IQR], 1.2 [0.9-1.7] vs 1.2 [1.0-1.7] mg/dL) and Elixhauser Comorbidity Index scores (mean [SD], 9.8 [9.6] vs 9.8 [9.9]) did not differ significantly between allergy groups. Patients with documented BL allergies incurred a higher average number of observed health care encounters (mean [SD], 12.5 [20.9] vs 10.6 [20.5] encounters) during the follow-up period, and a higher percentage of encounters with hemodialysis usage (mean [SD], 4.4% [20.6%] vs 4.2% [20.0%] encounters). The percentage of encounters that included an ICU admission (mean [SD], 10.6% [30.8%] vs 11.3% [31.6%]) was slightly higher in the non-BL-allergic group. The encounters occurred at 16 hospitals, with 3 hospitals (indicator numbers 13, 14, and 15), representing the largest number of encounters in both groups. The full demographics and characteristics can be seen in Table 1.

**Table 1.** Cohort Demographic and Clinical Characteristics

Characteristic^a^	Patients, No. (%)		*P* value
	Non–β-lactam allergic (n = 15 881)	β-lactam allergic (n = 4211)	
Age, mean (SD), y	62.6 (19.9)	64.2 (19.0)	<.001
Sex			
Male	6561 (41.3)	1300 (30.9)	<.001
Female	9320 (58.7)	2911 (69.1)	
Race			
Black	2849 (17.9)	664 (15.8)	<.001
White	12 026 (75.7)	3332 (79.1)	
Other^b^	1006 (6.3)	215 (5.1)	
Baseline serum creatinine, median (IQR), mg/dL	1.2 (1.0-1.7)	1.2 (0.9-1.7)	.94
Baseline estimated glomerular filtration rate, median (IQR), mL/min/1.73 m^2^	55.3 (35.6-70.6)	53.3 (34.0-69.5)	<.001
Elixhauser Comorbidity Index weighted summary score, mean (SD)	9.8 (9.9)	9.8 (9.6)	.99
Total No. of encounters^c^	168 327	52 607	NA
Health care encounters per patient over follow-up period, mean (SD), No.^c^	10.6 (20.5)	12.5 (20.9)	<.001
Encounters with intensive care unit admissions, mean (SD), %^c^	11.3 (31.6)	10.6 (30.8)	<.001
Encounters with hemodialysis usage, mean (SD), %^c^	4.2 (20.0)	4.4 (20.6)	.02
Hospital identifier for encounter			
1	417 (0.3)	149 (0.3)	<.001
2	1508 (0.9)	683 (1.3)	
3	4124 (2.5)	1363 (2.6)	
4	518 (0.3)	175 (0.3)	
5	477 (0.3)	318 (0.6)	
6	481 (0.3)	148 (0.3)	
7	101 (0.1)	15 (0.03)	
8	3385 (2.0)	977 (1.9)	
9	13 723 (8.2)	4197 (8.0)	
10	9968 (5.9)	3059 (5.8)	
11	782 (0.5)	204 (0.4)	
12	2940 (1.8)	989 (1.9)	
13	50 853 (30.2)	14 330 (27.2)	
14	44 497 (26.4)	14 862 (28.3)	
15	28 921 (17.2)	9640 (18.3)	
16	5632 (3.4)	1498 (2.9)	

SI conversion factor: To convert serum creatinine to micromoles per liter, multiply by 88.4.

^a^ Baseline characteristics were evaluated at first observed admission for each patient.

^b^ Other race refers to Aleutian-Indian, Asian, Chinese, Eskimo-Indian, Filipino, Hawaiian, Hispanic, Japanese, Middle Eastern, Oriental, Pacific Islander, multiracial, unknown, and other or declined.

^c^ Encounter-related data were calculated using information for the full follow-up period.

### Primary Outcome

The full unadjusted outcome counts can be seen in Table 2. The unadjusted mortality rate between allergy groups was higher in the BL-allergic group than in the group without BL allergy (9602 patients [60.5%] vs 2635 patients [62.6%]). There was not a significant difference in the time of death during follow-up, with a roughly equal proportion of deaths occurring within the first 2 years of follow-up (4648 patients [48.4%] without BL allergy vs 1261 patients [47.9%] with BL allergy) compared with the remaining 8 years (4954 patients [51.7%] without BL allergy vs 1374 patients [52.2%] with BL allergy). When the rate of mortality was compared using multivariable generalized estimating equations, patients with documented BL allergies did not experience a statistically significant difference in the odds of all-cause mortality compared with patients who did not have a documented BL allergy (OR, 1.02; 95% CI, 0.96-1.09). The full multivariable model results including secondary outcomes are shown in Table 3. When evaluating the full model, age, other race, increased Elixhauser Comorbidity Index score, and ICU encounters were significantly associated with an increase in all-cause mortality. The full model with all covariate effects is shown in eTable 2 in the Supplement.

**Table 2.** Outcome Counts by Allergy Status

Outcome	Patients, No. (%)^a^		*P* value
	Non–β-lactam allergic (n = 15 881)	β-lactam allergic (n = 4211)	
Death during study period	9602 (60.5)	2635 (62.6)	.01
Time to death, y			
0 to <1	3622 (37.7)	966 (36.7)	.72
1 to <2	1026 (10.7)	295 (11.2)	
2 to <5	2453 (25.6)	687 (26.1)	
5 to 10	2501 (26.1)	687 (26.1)	
MRSA infection	3870 (24.4)	1208 (28.7)	<.001
Time to first MRSA infection, y			
0 to <1	2412 (62.3)	756 (62.6)	.58
1 to <2	375 (9.7)	128 (10.6)	
2 to <5	612 (15.8)	192 (15.9)	
5 to 10	471 (12.2)	132 (10.9)	
No. of encounters with MRSA, mean (maximum)	0.43 (39)	0.57 (20)	<.001
*C difficile* infection	1393 (8.8)	388 (9.2)	.37
Time to first *C difficile* infection, y			
0 to <1	835 (60.0)	207 (52.4)	.13
1 to <2	116 (8.3)	38 (9.8)	
2 to <5	231 (16.6)	72 (18.6)	
5 to 10	211 (15.2)	71 (18.3)	
No. of encounters with *C difficile*, mean (maximum)	0.12 (17)	0.13 (8)	.18
VRE infection	1189 (7.5)	385 (9.1)	<.001
Time to first VRE infection, y			
0 to <1	784 (65.9)	239 (62.1)	.35
1 to <2	100 (8.4)	34 (8.8)	
2 to <5	192 (16.2)	64 (16.6)	
5 to 10	113 (9.5)	48 (12.5)	
No. of encounters with VRE, mean (maximum)	0.10 (17)	0.12 (8)	.009
Any antibiotic-resistant infection	5215 (32.8)	1549 (36.8)	<.001
Time to first antibiotic-resistant infection, y			
0 to <1	3427 (65.7)	1023 (66.0)	.71
1 to <2	450 (8.6)	141 (9.1)	
2 to <5	752 (14.4)	226 (14.6)	
5 to 10	586 (11.2)	159 (10.3)	
No. of encounters with antibiotic-resistant infection, mean (maximum)	0.61 (41)	0.77 (22)	<.001
Stage 2 and 3 AKI	4375 (27.6)	1161 (27.6)	.98
Time to first stage 2 and 3 AKI, y			
0 to <1	3173 (72.5)	799 (68.8)	.05
1 to <2	254 (5.8)	87 (7.5)	
2 to <5	489 (11.2)	143 (12.3)	
5 to 10	459 (10.5)	132 (11.4)	
No. of encounters with stage 2 and 3 AKI, mean (maximum)	0.79 (232)	0.96 (160)	.02
Stage 3 AKI	3164 (25.6)	843 (25.5)	.93
Time to first stage 3 AKI, y			
0 to <1	2282 (72.1)	574 (68.1)	.04
1 to <2	191 (6.0)	71 (8.4)	
2 to <5	351 (11.1)	103 (12.2)	
5 to 10	340 (10.8)	95 (11.3)	
No. of encounters with stage 3 AKI, mean (maximum)	0.69 (232)	0.87 (160)	.02

Abbreviations: AKI, acute kidney injury; *C difficile*, *Clostridium difficile*; MRSA, methicillin-resistant *Staphylococcus aureus*; VRE, vancomycin-resistant* Enterococcus*.

^a^ Outcomes are expressed as an indicator variable of occurrence vs not.

**Table 3.** Results of Generalized Estimation Equations^a^

Outcome	OR (95% CI) [95% CI using bootstrapped SEs]	*P* value
All-cause mortality	1.02 (0.96-1.09) [0.94-1.11]	.50
MRSA^b^	1.44 (1.36-1.53) [1.32-1.57]	<.001
*C difficile* ^b^	1.04 (0.94-1.16) [0.93-1.17]	.43
VRE^b^	1.18 (1.05-1.32) [1.05-1.32]	.004
Any resistant infection^c^	1.33 (1.30-1.36) [1.28-1.38]	<.001
Stage 2 to 3 AKI^d^	1.02 (0.96-1.10) [0.93-1.13]	.49
Stage 3 AKI^d^	1.06 (0.98-1.14) [0.95-1.17]	.15

Abbreviations: AKI, acute kidney injury; *C difficile*, *Clostridium difficile*; MRSA, methicillin-resistant *Staphylococcus aureus*; OR, odds ratio; VRE, vancomycin-resistant* Enterococcus*.

^a^ Only patient encounters with at least 1 serum creatinine level analyzed were included in the analysis (19 046 patients and 145 206 encounters).

^b^ Outcomes were not observed at all hospitals; 1% to 2% of the population could not be analyzed (MRSA, 20 092 patients and 220 303 encounters; *C difficile*, 20 092 patients and 219 621 encounters; and VRE, 20 092 patients and 219 494 encounters).

^c^ Any antibiotic-resistant infection is defined as pooled occurrence of MRSA, *C difficile*, or VRE.

^d^ The analysis controlled for age, race, sex, baseline serum creatinine level, number of health care encounters, Elixhauser Comorbidity Index score, dialysis utilization, intensive care unit admissions, and hospital location.

### Secondary Outcomes

Patients with documented BL allergies experienced higher rates of all antibiotic-resistant infection outcomes compared with the group without BL allergy, including MRSA (3870 patients [24.4%] vs 1208 patients [28.7%]), *C difficile* (1393 patients [8.8%] vs 388 patients [9.2%]), and VRE (1189 patients [7.5%] vs 385 patients [9.1%]). In addition, the unadjusted rate of any antibiotic-resistant infection was increased in the group with documented BL allergy vs the group without BL allergy (5215 patients [32.8%] vs 1549 patients [36.8%]). However, the unadjusted rate of stage 2 and 3 AKI (4375 patients [27.6%] vs 1161 patients [27.6%]) and stage 3 AKI (3164 patients [25.6%] vs 843 patients [25.5%]) did not differ between allergy groups.

Because secondary outcomes could occur more than once, we also evaluated the mean number of encounters per patient where each secondary outcome occurred, as well as the maximum number of each outcome experienced by a single patient in each allergy group. Similar to the overall percentage, the mean number of encounters with antibiotic-resistant infections was higher in the group with documented BL allergy, with MRSA (mean, 0.43 vs 0.57 encounters; maximum, 39 vs 20 encounters), *C difficile* (mean, 0.12 vs 0.13 encounters; maximum, 17 vs 8 encounters), VRE (mean, 0.10 vs 0.12 encounters; maximum, 17 vs 8 encounters), and pooled antibiotic-resistant infections (mean, 0.61 vs 0.77 encounters; maximum 41 vs 22 encounters) all being higher in the group of patients with documented BL allergies. In contrast to the overall binary occurrence, the mean number of encounters with AKI stage 2 and 3 AKI (mean, 0.96 vs 0.79 encounters; maximum, 232 vs 160 encounters) or stage 3 AKI (mean, 0.87 vs 0.69 encounters; maximum, 232 vs 160 encounters) per patient was higher in the BL-allergic group. In all instances, the maximum number of outcomes was higher in the group without documented BL allergies.

In the multivariable generalized estimating equations, the odds of MRSA (OR, 1.44; 95% CI, 1.36-1.53), VRE (OR, 1.18; 95% CI, 1.05-1.32), and pooled antibiotic-resistant infections (OR, 1.33; 95% CI, 1.30-1.36) were significantly associated with the presence of a documented BL allergy. There was no statistically significant difference in the rate of *C difficile* (OR, 1.04; 95% CI, 0.94-1.16), stage 2 and 3 AKI (OR, 1.02; 95% CI, 0.96-1.10), or stage 3 AKI (OR, 1.06; 95% CI, 0.98-1.14).

### Sensitivity Analysis

We performed 4 sensitivity analyses to further explore our results. First, when modeling documented BL allergy status as a dynamic variable (eTable 3 in the Supplement), documented BL allergy status contrasted with the effect seen using a static allergy status and was significantly associated with an increase in the odds of all-cause mortality (OR, 1.10; 95% CI, 1.07-1.17). The outcomes observed in the primary model with regard to the odds of infection with MRSA (OR, 1.56; 95% CI, 1.48-1.65), VRE (OR, 1.27; 95% CI, 1.14-1.42), and pooled antibiotic-resistant infections (OR, 1.36; 95% CI, 1.33-1.39) remained concordant with the primary model. However, when examining BL allergies as a dynamic variable evaluated at each encounter, documented BL allergies were significantly associated with VRE (OR, 1.27; 95% CI, 1.14-1.42), stage 2 and 3 AKI (OR, 1.07; 95% CI, 1.01-1.15), and stage 3 AKI (OR, 1.14; 95% CI, 1.06-1.23).

We then repeated the analysis while stratifying the cohort by whether a patient’s documented BL allergy status changed over follow-up, using both static and dynamic BL-allergy variables (eTables 4, 5, and 6 in Supplement 1). Documented BL allergies were more highly associated with all-cause mortality when examining only patients whose allergy status changed during follow-up; however, this represents a much smaller population than the full cohort, with only 955 patients having their allergy status change during follow-up. Analyzing only the patients whose allergy status did not change over follow-up did not appreciably change the results from the primary analysis. Next, we explored the potential bias introduced by imputing a relatively large (22%) percentage of baseline SCr values by repeating the analysis and not including baseline SCr as a covariate (eTable 7 in Supplement 1). All effect directions remained consistent when excluding baseline SCr as a covariate. We repeated the analysis while including LOS as a potential confounder. After including LOS as a covariate, the results did not appreciably change from those seen in the primary model (eTable 8 in Supplement 1).

## Discussion

This cohort study included 20 092 patients, of whom 4211 had documented BL allergy at the beginning of follow-up. Higher proportions of patients with documented BL allergies were older, female, and White race, which is consistent with demographics of previous allergy evaluations.^36,37,38^ Patients with documented BL allergies incurred higher rates of unadjusted all-cause mortality. When using generalized estimating equations to model clinical outcomes, the annual long-term adjusted odds of all-cause mortality was not significantly different in patients who had documented BL allergies compared with those who did not. We found that the risk for antibiotic-resistant infections was significantly increased in patients who had documented BL allergies, including increases of 44% and 18% in the odds for MRSA and VRE, respectively. However, we did not find statistically significant differences in the odds of stage 2 and 3 AKI or stage 3 AKI, or the odds of *C difficile* across BL allergy groups.

There has been only 1 previous set of long-term (>5 years follow-up) studies^13,14^ on the clinical outcomes of BL allergies, and they found a 14% increase in all-cause mortality and significant increases in the hazards of both MRSA and *C difficile*. Our results are similar to the previous results in terms of finding significant associations between documented BL allergies and an increase in MRSA infection, but our results also found a significant association of VRE infection with documented BL allergies, as well as the risk of the pooled rate of infection with MRSA, *C difficile*, and VRE. In contrast, our primary results did not find a significant difference in all-cause mortality associated with documented BL allergies. However, we did find a significant association of all-cause mortality with documented BL allergy status when using a time-varying allergy status, so the long-term association of documented BL allergy status with all-cause mortality remains unclear. Compared with the previous studies by Blumenthal et al,^13,14^ our study provides a unique perspective using data from a different region and the consideration of documented BL allergy status to act as a dynamic variable. Although a relatively small amount of our population experienced a change in their documented BL allergy status during follow-up, the association of documented BL allergies with mortality was greater when modeling allergy status in a dynamic manner, and future studies should further evaluate whether changes in a patient’s documented BL allergy status over time affect the care they receive.

Our study also extends upon these previously completed cohort studies by analyzing an additional resistant infection of VRE, as well as evaluating the long-term association of documented BL allergies with AKI, which to the best of our knowledge, has not been previously described. Previous shorter-term studies^8,39,40^ have examined the association of documented BL allergies with AKI, with inconsistent results that have often not met statistical significance. Our results also did not find a statistically significant association of documented BL allergies with either stage 2 and 3 AKI or stage 3 AKI, but did find a significant association when modeling documented BL allergy status as a dynamic variable. Our analysis was limited by only using SCr level to evaluate AKI, and we were only able to evaluate health care encounters where at least 1 SCr measurement was present. Future studies should aim to improve upon the understanding of the long-term association of BL allergies with AKI by including additional laboratory values and attempting to more directly link changes in prescribing practices to AKI.

The long-term clinical outcomes of BL allergies are understudied, and our study addressed this gap in the literature by being uniquely designed to evaluate the long-term clinical outcomes associated with BL allergies in a novel population.^12^ Shorter-term studies^41,42^ have not consistently found an association between BL allergies and mortality. However, longitudinal evaluations such as the results of our study using a time-varying allergy status and the previous cohort studies conducted by Blumenthal et al^13,14^ have more consistently identified potential positive associations of BL allergies with increased mortality, and this may indicate that the mortality-related effects associated with BL allergies become more apparent over an extended time.^14^ Future studies should further explore the long-term effect of documented BL allergy status and mortality, as well as expand the understanding of the long-term effects of BL allergy delabeling by comparing the outcomes for patients who had BL allergies delabeled.

### Limitations

Although our study had an overall large sample size of more than 20 000 patients, it is smaller than previous cohort studies examining similar outcomes.^14^ Overall, our study used data from a single regionally based health care system in western Pennsylvania and may not apply broadly outside this region. The definition used to define AKI was limited to changes in SCr alone, which is further complicated by the lack of evidence-supported consensus methods for the establishment of baseline SCr, but is the first attempt to relate long-term effects of BL allergies to kidney-related outcomes.^31^ Our analysis was not able to evaluate whether the cause of death was related to infectious causes, which would be valuable to understanding whether documented BL allergies have a differential effect on these causes of death. Our population was also limited to patients with a diagnosis of sepsis, pneumonia, and UTI between a 2-year span, and although limiting our cohort to these diagnoses reduced the risk for immortal time bias and indication bias, it reduces the external generalizability and sample size of our results. In addition, we were unable to account for the severity of particular BL allergies, which would be of great interest to examining whether more severe reactions are underlying the observed higher rates of mortality and resistant infections due to necessitating a higher level of change in clinical practice than less severe allergies.

## Conclusions

In this 12-year longitudinal analysis, we used generalized estimating equations to evaluate the long-term clinical consequences of patients having a BL allergy documented in their EHR. The presence of a documented BL allergy was not associated with a statistically significant difference in the odds of all-cause mortality compared with patients without a documented BL allergy. Documented BL allergies were associated with increased odds of antibiotic-resistant infections, including MRSA, VRE, and the pooled odds of infection with MRSA, VRE, and *C difficile*. Health systems should support accurate allergy labeling and reduce the risk of unnecessary BL avoidance to improve patient outcomes.

## Notes

Supplement 1. **eTable 1.** Definition of Pneumonia, UTI and Sepsis Using *ICD-9 *Codes **eTable 2.** Full Model Summary of Generalized Estimating Equation Predicting All-Cause Mortality **eTable 3.** Results of Generalized Estimating Equations Using Dynamic Allergy Status **eTable 4.** Results Among Patients Whose β-Lactam Allergy Status Changed During Follow-up (Initial Allergy Status Held Constant) **eTable 5.** Results Among Patients Whose β-Lactam Allergy Status Changed During Follow-up (Time-Varying Allergy Status) **eTable 6.** Results among Patients Whose β-Lactam Allergy Status Did Not Change During Follow-up **eTable 7.** Results of Generalized Estimating Equations Sensitivity Analysis Excluding Baseline Serum Creatinine as a Covariate **eTable 8.** Results of Generalized Estimating Equations Sensitivity Analysis Including Length of Stay

Supplement 2.
Data Sharing Statement
