# Supplementary material for: Long-Term Outcomes Associated With β-Lactam Allergies
Source: JAMA Netw Open. 2024 May 17;7(5):e2412313. doi: 10.1001/jamanetworkopen.2024.12313 (PMC11102016; doi:10.1001/jamanetworkopen.2024.12313)
Supplement: Supplement 1. — eTable 1. Definition of Pneumonia, UTI and Sepsis Using ICD-9 Codes eTable 2. Full Model Summary of Generalized Estimating Equation Predicting All-Cause Mortality eTable 3. Results of Generalized Estimating Equations Using Dynamic Allergy Status eTable 4. Results Among Patients Whose β-Lactam Allergy Status Changed During Follow-up (Initial Allergy Status Held Constant) eTable 5. Results Among Patients Whose β-Lactam Allergy Status Changed During Follow-up (Time-Varying Allergy Status) eTable 6. Results among Patients Whose β-Lactam Allergy Status Did Not Change During Follow-up eTable 7. Results of Generalized Estimating Equations Sensitivity Analysis Excluding Baseline Serum Creatinine as a Covariate eTable 8. Results of Generalized Estimating Equations Sensitivity Analysis Including Length of Stay [file jamanetwopen-e2412313-s001.pdf]

Supplemental Online Content

Gray MP, Kellum JA, Kirisci L, Boyce RD, Kane-Gill SL. Long-term outcomes associated with β-lactam allergies. *JAMA Netw Open*. 2024;7(5):e2412313.
doi:10.1001/jamanetworkopen.2024.12313

eTable 1. Definition of Pneumonia, UTI and Sepsis Using *ICD-9* Codes

eTable 2. Full Model Summary of Generalized Estimating Equation Predicting All-Cause Mortality

eTable 3. Results of Generalized Estimating Equations Using Dynamic Allergy Status

eTable 4. Results Among Patients Whose β-Lactam Allergy Status Changed During Follow-up (Initial Allergy Status Held Constant)

eTable 5. Results Among Patients Whose β-Lactam Allergy Status Changed During Follow-up (Time-Varying Allergy Status)

eTable 6. Results among Patients Whose β-Lactam Allergy Status Did Not Change During Follow-up

eTable 7. Results of Generalized Estimating Equations Sensitivity Analysis Excluding Baseline Serum Creatinine as a Covariate

eTable 8. Results of Generalized Estimating Equations Sensitivity Analysis Including Length of Stay

This supplemental material has been provided by the authors to give readers additional information about their work.

© 2024 Gray MP et al. *JAMA Network Open*.

eTable 1: Definition of Pneumonia, UTI and Sepsis Using *ICD-9* Codes

ICD-9 Codes Indicating Pneumonia	480.0, 480.1, 480.2, 480.3, 480.8, 480.9, 481, 482.0, 482.1, 482.2, 482.30, 482.31, 482.32, 482.39, 482.40, 482.41, 482.49, 482.81, 482.82, 482.83, 482.84, 482.89, 482.9, 483.0, 483.1, 483.8, 484.1, 484.3, 484.5, 484.6, 484.7, 484.8, 485, 486, 487.0
ICD-9 Codes Indicating Urinary Tract Infection	599.0

	Code stem	Included codes	Code stem meaning
ICD-9 codes indicating infection	001	001.0, 001.1, 001.9	Cholera
	002	002.0, 002.1, 002.2, 002.3, 002.9	Typhoid/paratyphoid fever
	003	003.0, 003.1, 003.20, 003.21, 003.22, 003.23, 003.24, 003.29, 003.8, 003.9	Other salmonella infection
	004	004.0, 004.1, 004.2, 004.3, 004.8, 004.9	Shigellosis
	005	005.0, 005.1, 005.2, 005.3, 005.4, 005.81, 005.89, 005.9	Other food poisoning
	008	008.00, 008.01, 008.02, 008.03, 008.04, 008.09, 008.1, 008.2, 008.3, 008.41, 008.42, 008.43, 008.44, 008.45, 008.46, 008.47, 008.49, 008.5, 008.61, 008.62, 008.63, 008.64, 008.65, 008.66, 008.67, 008.69, 008.8	Intestinal infection not otherwise classified
	009	009.0, 009.1, 009.2, 009.3	Ill-defined intestinal infection
	010	010.00, 010.01, 010.02, 010.03, 010.04, 010.05, 010.06, 010.10, 010.11, 010.12, 010.13, 010.14, 010.15, 010.16, 010.80, 010.81, 010.82, 010.83, 010.84, 010.85, 010.86, 010.90, 010.91, 010.92, 010.93, 010.94, 010.95, 010.96	Primary tuberculosis infection
	011	011.00, 011.01, 011.02, 011.03, 011.04, 011.05, 011.06, 011.10, 011.11, 011.12, 011.13, 011.14, 011.15, 011.16, 011.20, 011.21, 011.22, 011.23, 011.24, 011.25, 011.26, 011.30, 011.31, 011.32, 011.33, 011.34, 011.35, 011.36, 011.40, 011.41, 011.42, 011.43, 011.44, 011.45, 011.46, 011.50, 011.51, 011.52, 011.53, 011.54, 011.55, 011.56, 011.60, 011.61, 011.62, 011.63, 011.64, 011.65, 011.66, 011.70, 011.71, 011.72, 011.73, 011.74, 011.75, 011.76, 011.80, 011.81, 011.82, 011.83, 011.84, 011.85, 011.86, 011.90, 011.91, 011.92, 011.93, 011.94, 011.95, 011.96	Pulmonary tuberculosis
	012	012.00, 012.01, 012.02, 012.03, 012.04, 012.05, 012.06, 012.10, 012.11, 012.12, 012.13, 012.14, 012.15, 012.16, 012.20, 012.21, 012.22, 012.23, 012.24, 012.25,	Other respiratory tuberculosis

© 2024 Gray MP et al. *JAMA Network Open*.

		012.26, 012.30, 012.31, 012.32, 012.33, 012.34, 012.35, 012.36, 012.80, 012.81, 012.82, 012.83, 012.84, 012.85, 012.86	
	013	013.00, 013.01, 013.02, 013.03, 013.04, 013.05, 013.06, 013.10, 013.11, 013.12, 013.13, 013.14, 013.15, 013.16, 013.20, 013.21, 013.22, 013.23, 013.24, 013.25, 013.26, 013.30, 013.31, 013.32, 013.33, 013.34, 013.35, 013.36, 013.40, 013.41, 013.42, 013.43, 013.44, 013.45, 013.46, 013.50, 013.51, 013.52, 013.53, 013.53, 013.54, 013.55, 013.56, 013.60, 013.61, 013.62, 013.63, 013.64, 013.65, 013.66, 013.80, 013.81, 013.82, 013.83, 013.84, 013.85, 013.86, 013.90, 013.91, 013.92, 013.93, 013.94, 013.95, 013.96	Central nervous system tuberculosis
	014	014.00, 014.01, 014.02, 014.03, 014.04, 014.05, 014.06, 014.80, 014.81, 014.82, 014.83, 014.84, 014.85, 014.86	Intestinal tuberculosis
	015	015.00, 015.01, 015.02, 015.03, 015.04, 015.05, 015.06, 015.10, 015.11, 015.12, 015.13, 015.14, 015.15, 015.16, 015.20, 015.21, 015.22, 015.23, 015.24, 015.25, 015.26, 015.50, 015.51, 015.52, 015.53, 015.54, 015.55, 015.56, 015.60, 015.61, 015.62, 015.63, 015.64, 015.65, 015.66, 015.70, 015.71, 015.72, 015.73, 015.74, 015.75, 015.76, 015.80, 015.81, 015.82, 015.83, 015.84, 015.85, 015.86, 015.90, 015.91, 015.92, 015.93, 015.94, 015.95, 015.96	Tuberculosis of bone and joint
	016	160.00, 016.01, 016.02, 016.03, 016.04, 016.05, 016.06, 016.10, 016.11, 016.12, 016.13, 016.14, 016.15, 016.16, 016.20, 016.21, 016.22, 016.23, 016.24, 016.25, 016.26, 016.30, 016.31, 016.32, 016.33, 016.34, 016.35, 016.36, 016.40, 016.41, 016.42, 016.42, 016.43, 016.44, 016.45, 016.46, 016.50, 016.51, 016.52, 016.53, 016.54, 016.55, 016.56, 016.60, 016.61, 016.62, 016.63, 016.64, 016.65, 016.66, 016.70, 016.71, 016.72, 016.73, 016.74, 016.75, 016.76, 016.90, 016.91, 016.92, 016.93, 016.94, 016.95, 016.96	Genitourinary tuberculosis
	017	017.00, 017.01, 017.02, 017.03, 017.04, 017.05, 017.06, 017.10, 017.11, 017.12, 017.13, 017.14, 017.15, 017.16, 017.20, 017.21, 017.22, 017.23, 017.24, 017.25, 017.26, 017.30, 017.31, 017.32, 017.33, 017.34, 017.35, 017.36, 017.40, 017.41, 017.42, 017.43, 017.44, 017.45, 017.46, 017.50, 017.51, 017.52, 017.53, 017.54, 017.55, 017.56, 017.60, 017.61, 017.62, 017.63, 017.64, 017.65, 017.66, 017.70, 017.71, 017.72, 017.73, 017.74, 017.75,	Tuberculosis not otherwise classified

© 2024 Gray MP et al. *JAMA Network Open*.

		017.76, 017.80, 017.81, 017.82, 017.83, 017.84, 017.85, 017.86, 017.90, 017.91, 017.92, 017.93, 017.94, 017.95, 017.96	
	018	018.00, 018.01, 018.02, 018.03, 018.04, 018.05, 018.06, 018.80, 018.81, 018.82, 018.83, 018.84, 018.85, 018.86, 018.90, 018.91, 018.92, 018.93, 018.94, 018.95, 018.96	Miliary tuberculosis
	020	020.0, 020.1, 020.2, 020.3, 020.4, 020.5, 020.8, 020.9	Plague
	021	021.0, 021.1, 021.2, 021.3, 021.8, 021.9	Tularemia
	022	022.0, 022.1, 022.2, 022.3, 022.8, 022.9	Anthrax
	023	023.0, 023.1, 023.2, 023.8, 023.9	Brucellosis
	024	024	Glanders
	025	025	Melioidosis
	026	026.0, 026.1, 026.9	Rat-bite fever
	027	027.0, 027.1, 027.2, 027.8, 027.9	Other bacterial zoonoses
	030	030.0, 030.1, 030.2, 030.3, 030.8, 030.9	Leprosy
	031	031.0, 031.1, 031.2, 031.8, 031.9	Other mycobacterial disease
	032	032.0, 032.1, 032.3, 032.81, 032.82, 032.83, 032.84, 032.85, 032.89, 032.9	Diphtheria
	033	033.0, 033.1, 033.8, 033.9	Whooping cough
	034	034.0, 034.1	Streptococcal throat/scarlet fever
	035	035	Erysipelas
	036	036.0, 036.1, 036.2, 036.3, 036.40, 036.41, 036.42, 036.43, 036.81, 036.82, 036.89, 036.9	Meningococcal infection
	037	037	Tetanus
	038	038.0, 038.10, 038.11, 038.19, 038.2, 038.3, 038.40, 038.41, 038.42, 038.43, 038.44, 038.49, 038.8, 038.9	Septicemia
	039	039.0, 039.1, 039.2, 039.3, 039.4, 039.8, 039.9	Actinomycotic infections
	040	040.0, 040.1, 040.2, 040.3, 040.41, 040.42, 040.81, 040.82, 040.89	Other bacterial diseases
	041	041.00, 041.01, 041.02, 041.03, 041.04, 041.05, 041.09, 041.10, 041.11, 041.19, 041.2, 041.3, 041.4, 041.5, 041.6, 041.7, 041.81, 041.82, 041.83, 041.84, 041.85, 041.86, 041.89, 041.9	Bacterial infection in other diseases not otherwise specified
	090	090.0, 090.1, 090.2, 090.3, 090.40, 090.41, 090.42. 090.49, 090.5, 090.6, 090.7, 090.9	Congenital syphilis
	091	091.0, 091.1, 091.2, 091.3, 091.4, 091.50, 091.51, 091.52, 091.61, 091.62, 091.69, 091.7, 091.81, 091.82, 091.89, 091.9	Early symptomatic syphilis
	092	092.0, 092.9	Early syphilis latent
	093	093.0, 093.1, 093.20, 093.21, 093.22, 093.23, 093.24, 093.81, 093.82, 093.89, 093.9	Cardiovascular syphilis

© 2024 Gray MP et al. *JAMA Network Open*.

	094	094.0, 094.1, 094.2, 094.3, 094.81, 094.82, 094.83, 094.84, 094.85, 094.86, 094.87, 094.89, 094.9	Neurosyphilis
	095	095.0, 095.1, 095.2, 095.3, 095.4, 095.5, 095.6, 095.7, 095.8, 095.9	Other late symptomatic syphilis
	096	096	Late syphilis latent
	097	097.0, 097.1, 097.9	Other and unspecified syphilis
	098	098.0, 098.10, 098.11, 098.12, 098.13, 098.14, 098.15, 098.16, 09.17, 098.19, 098.2, 098.30, 098.31, 098.32, 098.33, 098.34, 098.35, 098.36, 098.37, 098.39, 098.40, 098.41, 098.42, 098.43, 098.49, 098.50, 098.51, 098.52, 098.53, 098.59, 098.6, 098.7, 098.81, 098.82, 098.83, 098.84, 098.85, 098.86, 098.89	Gonococcal infections
	100	100.0, 100.81, 100.89, 100.9	Leptospirosis
	101	101	Vincent’s angina
	102	102.0, 102.1, 102.2, 102.3, 102.4, 102.5, 102.6, 102.7, 102.8, 102.9	Yaws
	103	103.0, 103.1, 103.2, 103.3, 103.9	Pinta
	104	104.0, 104.8, 104.9	Other spirochetal infection
	110	110.0, 110.1, 110.2, 110.3, 110.4, 110.5, 110.6, 110.8, 110.9	Dermatophytosis
	111	111.0, 111.1, 111.2, 111.3, 111.8, 111.9	Dermatomycosis not otherwise classified or specified
	112	112.0, 112.1, 112.2, 112.3, 112.4, 112.5, 112.81, 112.82, 112.83, 112.84, 112.85, 112.89, 112.9	Candidiasis
	114	114.0, 114.1, 114.2, 114.3, 114.4, 114.5, 114.9	Coccidioidomycosis
	115	115.00, 115.01, 115.02, 115.03, 115.04, 115.05, 115.06, 115.10, 115.11, 115.12, 115.13, 115.14, 115.15, 115.19, 115.90, 115.91, 115.92, 115.93, 115.94, 115.95, 115.99	Histoplasmosis
	116	116.0, 116.1, 116.2	Blastomycotic infection
	117	117.0, 117.1, 117.2, 117.3, 117.4, 117.5, 117.6, 117.7, 117.8, 117.9	Other mycoses
	118	118	Opportunistic mycoses
	320	320.0, 320.1, 320.2, 320.3, 320.7, 320.81, 320.82, 320.89, 320.9	Bacterial meningitis
	322	322.0, 322.1, 322.2, 322.9	Meningitis
	324	324.0, 324.1, 324.9	Central nervous system abscess
	325	325	Phlebitis of intracranial sinus
	420	420.0, 420.90, 420.91, 420.99	Acute pericarditis
	421	421.0, 421.1, 421.9	Acute or subacute endocarditis
	451	451.0, 451.11, 451.19, 451.2, 451.81, 451.82, 451.83, 451.84, 451.89, 451.9	Thrombophlebitis
	461	461.0, 461.1, 461.2, 461.3, 461.8, 461.9	Acute sinusitis
	462	462	Acute pharyngitis

© 2024 Gray MP et al. *JAMA Network Open*.

	463	463	Acute tonsillitis
	464	464.00, 464.01, 464.10, 464.11, 464.20, 464.21, 464.30, 464.31, 464.4, 464.50, 464.51	Acute laryngitis/tracheitis
	465	465.0, 465.8, 465.9	Acute upper respiratory infection of multiple sites/not otherwise specified
	481	481	Pneumococcal pneumonia
	482	482.0, 482.1, 482.30, 482.31, 482.32, 482.39, 482.40, 482.41, 482.49, 482.81, 482.82, 482.83, 482.84, 482.89, 482.9	Other bacterial pneumonia
	485	485	Bronchopneumonia with organism not otherwise specified
	486	486	Pneumonia
	491.21	491.21	Acute exacerbation of obstructive chronic bronchitis
	494	494.0, 494.1	Bronchiectasis
	510	510.0, 510.9	Empyema
	513	513.0, 513.1	Lung/mediastinum abscess
	540	540.0, 540.1, 540.9	Acute appendicitis
	541	541	Appendicitis not otherwise specified
	542	542	Other appendicitis
	562.01	562.01	Diverticulitis of small intestine without hemorrhage
	562.03	562.03	Diverticulitis of small intestine with hemorrhage
	562.11	562.11	Diverticulitis of colon without hemorrhage
	562.13	562.13	Diverticulitis of colon with hemorrhage
	566	566	Anal and rectal abscess
	567	567.0, 567.1, 567.21, 567.22, 567.23, 567.29, 567.31, 567.38, 567.39, 567.81, 567.82, 567.89, 567.9	Peritonitis
	569.5	569.5	Intestinal abscess
	569.83	569.83	Perforation of intestine
	572.0	572.0	Abscess of liver
	572.1	572.1	Portal pyemia
	575	575.0, 575.10, 575.11, 575.12, 575.2, 575.3, 575.4, 575.5, 575.6, 575.8, 575.9	Acute cholecystitis
	590	590.00, 590.01, 590.10, 590.11, 590.2, 590.3, 590.80, 590.81, 590.9	Kidney infection
	597	597.0, 597.80, 597.81, 597.89	Urethritis/ urethral syndrome
	599	599.0, 599.1, 599.2, 599.3, 599.4, 599.5, 599.60, 599.61, 599.7, 599.81, 599.82, 599.83, 599.84, 599.89, 599.9	Urinary tract infection not otherwise specified
	601	601.0, 601.1, 601.2, 601.3, 601.4, 601.8, 601.9	Prostatic inflammation
	614	614.0, 614.1, 614.2, 614.3, 614.4, 614.5, 614.6, 614.7, 614.8, 614.9	Female pelvic inflammation disease

© 2024 Gray MP et al. *JAMA Network Open*.

	615	615.0, 615.1, 615.9	Uterine inflammatory disease
	616	616.0, 616.10, 616.11, 616.2, 616.3, 616.4, 616.50, 616.51, 616.81, 616.89, 616.9	Other female genital inflammation
	681	681.00, 681.01, 681.02, 681.10, 681.11, 681.9	Cellulitis
	682	682.0, 682.1, 682.2, 682.3, 682.4, 682.5, 682.6, 682.7, 682.8, 682.9	Other cellulitis or abscess
	683	683	Acute lymphadenitis
	686	686.00, 686.01, 686.09, 686.1, 686.8, 686.9	Other local skin infection
	711	711.00, 711.01, 711.02, 711.03, 711.04, 711.05, 711.06, 711.07, 711.08, 711.09, 711.10, 711.11, 711.12, 711.13, 711.14, 711.15, 711.16, 711.17, 711.18, 711.19, 711.20, 711.21, 711.22, 711.23, 711.24, 711.25, 711.26, 711.27, 711.28, 711.29, 711.30, 711.31, 711.32, 711.33, 711.34, 711.35, 711.36, 711.37 711.38, 711.39, 711.40, 711.41, 711.42, 711.43, 711.44, 711.45, 711.46, 711.47, 711.48, 711.49, 711.50, 711.51, 711.52, 711.53, 711.54, 711.55, 711.56, 711.57, 711.58, 711.59, 711.60, 711.61, 711.62, 711.63, 711.64, 711.65, 711.66, 711.67, 711.68, 711.69, 711.70, 711.71, 711.72, 711.73, 711.74, 711.75, 711.76, 711.77, 711.78, 711.79, 711.80, 711.81, 711.82, 711.83, 711.84, 711.85, 711.86, 711.87, 711.88, 711.89, 711.90, 711.91, 711.92, 711.93, 711.94, 711.95, 711.96, 711.97, 711.98, 711.99	Pyogenic arthritis
	730	730.00, 730.01, 730.02, 730.03, 730.04, 730.05, 730.06, 730.07, 730.08, 730.09, 730.10, 730.11, 730.12, 730.13, 730.14, 730.15, 730.16, 730.17, 730.18, 730.19, 730.20, 730.21, 730.22, 730.23, 730.24, 730.25, 730.26, 730.27, 730.28, 730.29, 730.30, 730.31, 730.32, 730.33, 730.34, 730.35, 730.36, 730.37, 730.38, 730.39, 730.70, 730.71, 730.72, 730.73, 730.74, 730.75, 730.76, 730.77, 730.78, 730.79, 730.80, 730.81, 730.82, 730.83, 730.84, 730.85, 730.86, 730.87, 730.88, 730.89, 730.90, 730.91, 730.92, 730.93, 730.94, 730.95, 730.96, 730.97, 730.98, 730.99	Osteomyelitis
	790.7	790.7	Bacteremia
	996.6	996.61, 996.62, 996.63, 996.64, 996.65, 996.66, 996.67, 996.68, 996.69	Infection or inflammation of device/graft
	998.5	998.51, 998.59	Postoperative infection
	999.3	999.31, 999.39	Infectious complication of medical care not otherwise classified.
ICD-9 codes indicating	785.5	785.50, 785.51, 785.52, 785.59	shock without trauma
	458	458.0, 458.1, 458.21, 458.29, 458.8, 458.9	hypotension

organ damage		348.3	348.30, 348.31, 348.39	encephalopathy
		293	293.0, 293.1, 293.81, 293.82, 293.83, 293.84, 293.89, 293.9	transient organic psychosis
		348.1	348.1	anoxic brain damage
		287.4	287.4	secondary thrombocytopenia
		287.5	287.5	thrombocytopenia, unspecified
		286.6	286.6	defibrination syndrome
		286.9	286.9	other/unspecified coagulation defect
		570	570	acute and subacute necrosis of liver
		573.4	573.4	hepatic infarction
		584	584.5, 584.6, 584.7, 584.8, 584.9	acute renal failure

© 2024 Gray MP et al. *JAMA Network Open*.

eTable 2: Full Model Summary of Generalized Estimating Equation Predicting All-Cause Mortality

Covariate	Odds Ratio (95% CI)	p-value
**β-Lactam allergy**	1.02 (0.96 – 1.09)	0.495
**Female Sex**	0.82 (0.78 – 0.87)	<0.001
**Age**	1.05 (1.05 – 1.05)	<0.001
**Race:***		
**Black**	0.93 (0.86 – 1.00)	0.061
**Other**	1.23 (1.08 – 1.41)	0.002
**Baseline SCr**	0.86 (0.85 – 0.88)	<0.001
**Elixhauser (Van-Walraven) Comorbidity Index**	1.10 (1.10 – 1.10)	<0.001
**Dialysis during Encounter**	6.63 (5.93 – 7.43)	<0.001
**Intensive Care Encounter**	5.25 (4.93 – 5.60)	<0.001
**Number of Total Encounters**	0.94 (0.94 – 0.94)	<0.001
**Hospital Number:**		
**1**	0.32 (0.20 – 0.53)	<0.001
**2**	0.54 (0.33 – 0.86)	0.010
**3**	0.31 (0.16 – 0.59)	<0.001
**4**	0.26 (0.13 – 0.53)	<0.001
**5**	0.68 (0.34 – 1.33)	0.26
**6**	0.87 (0.26 – 2.88)	0.814
**7**	0.40 (0.24 – 0.66)	<0.001
**8**	0.45 (0.29 – 0.71)	0.001
**9**	0.17 (0.11 – 0.27)	<0.001
**10**	0.48 (0.27 – 0.87)	0.016
**11**	0.53 (0.33 – 0.85)	0.008
**12**	0.30 (0.19 – 0.47)	<0.001
**13**	0.29 (0.19 – 0.45)	<0.001
**14**	0.27 (0.18 – 0.43)	<0.001
**15**	0.16 (0.10 – 0.25)	<0.001

© 2024 Gray MP et al. *JAMA Network Open*.

eTable 3: Results of Generalized Estimating Equations Using Dynamic Allergy Status

Outcome	Odds Ratio (95% CI)	p-value
All-cause mortality	1.10 (1.03 – 1.17)	0.002
MRSA[a]	1.56 (1.48 – 1.65)	<0.001
CDiff[a]	1.14 (1.03 – 1.26)	0.012
VRE[a]	1.27 (1.14 – 1.42)	<0.001
Any resistant infection[a,b]	1.36 (1.33 – 1.39)	<0.001
Stage 2/3 AKI[c]	1.07 (1.01 – 1.15)	0.033
Stage 3 AKI[c]	1.14 (1.06 – 1.23)	<0.001

Legend: Controlled for: age, race, sex, baseline serum creatinine, number of healthcare encounters, Elixhauser comorbidity score, dialysis utilization, intensive care admissions, and hospital location.

a. Outcomes were not observed at all hospitals; between 1-2% of the population could not be analyzed (MRSA: 20,092 patients, 220,303 encounters; CDiff: 20,092 patients, 219,621 encounters; VRE: 20,092 patients, 219,494 encounters)
b. Any resistant infection defined as pooled occurrence of MRSA, CDiff, or VRE

Only patient encounters with at least one SCr analyzed (19,046 patients; 145206 encounters)

© 2024 Gray MP et al. *JAMA Network Open*.

eTable 4: Results Among Patients Whose β-Lactam Allergy Status Changed During Follow-up (Initial Allergy Status Held Constant)

Outcome	Odds Ratio (95% CI)	p-value
**All-cause mortality**[a]	0.92 (0.68 – 1.24)	0.582
**MRSA**[a]	1.01 (0.83 – 1.24)	0.889
**CDiff**[a]	1.14 (0.76 – 1.71)	0.517
**VRE**[a]	1.05 (0.70 – 1.57)	0.818
**Any Resistant Infection**[a,b]	1.06 (0.89 – 1.27)	0.499
**Stage 2/3 AKI**[c]	1.00 (0.76 – 1.33)	0.975
**Stage 3 AKI**[d]	1.05 (0.78 – 1.42)	0.735

Legend: Controlled for: age, race, sex, baseline serum creatinine, number of healthcare encounters, Elixhauser comorbidity score, dialysis utilization, intensive care admissions and hospital location. N=21,183 encounters; 955 patients

a. Outcomes were not observed at all hospitals; between 1-2% of the population could not be analyzed (All-cause Mortality: 955 patients; 20,955 encounters; MRSA: 955 patients, 21,126 encounters; CDiff: 938 patients, 19,780 encounters; VRE: 953 patients, 20,207 encounters; Any Resistant Infection: 955 patients; 21,126 encounters)
b. Any resistant infection defined as pooled occurrence of MRSA, CDiff, or VRE
c. Outcomes were not observed at all hospitals; Only patient encounters with at least one SCr analyzed (918 patients; 14,035 encounters)
d. Outcomes were not observed at all hospitals; Only patient encounters with at least one SCr analyzed (908 patients; 13,837 encounters)

© 2024 Gray MP et al. *JAMA Network Open*.

eTable 5: Results Among Patients Whose β-Lactam Allergy Status Changed During Follow-up (Time-Varying Allergy Status)

Outcome	Odds Ratio (95% CI)	p-value
**All-cause mortality**	6.02 (4.51 – 8.03)	<0.001
**MRSA**[a]	1.30 (1.11 – 1.53)	0.001
**CDiff**[a]	1.37 (0.99 – 1.90)	0.060
**VRE**[a]	1.10 (0.80 – 1.52)	0.559
**Any Resistant Infection**[a,b]	1.34 (1.16 – 1.55)	<0.001
**Stage 2/3 AKI**[c]	1.67 (1.35 – 2.08)	<0.001
**Stage 3 AKI**[d]	1.85 (1.46 – 2.34)	<0.001

Legend: Controlled for: age, race, sex, baseline serum creatinine, number of healthcare encounters, Elixhauser comorbidity score, dialysis utilization, intensive care admissions and hospital location. N=21,183 encounters; 955 patients

a. Outcomes were not observed at all hospitals; between 1-2% of the population could not be analyzed (All-cause Mortality: 955 patients; 20,955 encounters; MRSA: 955 patients, 21,126 encounters; CDiff: 938 patients, 19,780 encounters; VRE: 953 patients, 20,207 encounters; Any Resistant Infection: 955 patients; 21,126 encounters)
b. Any resistant infection defined as pooled occurrence of MRSA, CDiff, or VRE
c. Outcomes were not observed at all hospitals; Only patient encounters with at least one SCr analyzed (918 patients; 14,035 encounters)
d. Outcomes were not observed at all hospitals; Only patient encounters with at least one SCr analyzed (908 patients; 13,837 encounters)

© 2024 Gray MP et al. *JAMA Network Open*.

eTable 6. Results among Patients Whose β-Lactam Allergy Status Did Not Change During Follow-up

Outcome	Odds Ratio (95% CI)	p-value
**All-cause mortality**	1.02 (0.96 – 1.09)	0.516
**MRSA[a]**	1.50 (1.41 – 1.59)	<0.001
**CDiff[a]**	1.04 (0.93 – 1.16)	0.45
**VRE[a]**	1.19 (1.06 – 1.33)	0.004
**Any Resistant Infection[a,b]**	1.39 (1.31 – 1.47)	<0.001
**Stage 2/3 AKI[c]**	1.03 (0.96 – 1.10)	0.487
**Stage 3 AKI[c]**	1.06 (0.98 – 1.15)	0.144

Legend: Controlled for: age, race, sex, baseline serum creatinine, number of healthcare encounters, Elixhauser comorbidity score, dialysis utilization, intensive care admissions and hospital location. N=199,750 encounters; 19,137 patients

a. Outcomes were not observed at all hospitals; between 1-2% of the population could not be analyzed (All-cause Mortality: 19,137 patients; 199,750 encounters; MRSA: 19,137 patients, 199,177 encounters; CDiff: 19,137 patients, 198,600 encounters; VRE: 19,137 patients, 197,658 encounters; Any Resistant Infection: 19,137 patients; 199,177 encounters)
b. Any resistant infection defined as pooled occurrence of MRSA, CDiff, or VRE
c. Outcomes were not observed at all hospitals; Only patient encounters with at least one SCr analyzed (18,128 patients; 131,008 encounters)

© 2024 Gray MP et al. *JAMA Network Open*.

eTable 7: Results of Generalized Estimating Equations Sensitivity Analysis Excluding Baseline Serum Creatinine as a Covariate

Outcome	Odds Ratio (95% CI)	p-value
All-cause mortality	1.03 (0.97 – 1.10)	0.376
MRSA[a]	1.45 (1.37 – 1.54)	<0.001
CDiff[a]	1.06 (0.96 – 1.18)	0.287
VRE[a]	1.19 (1.07 – 1.33)	0.002
Any resistant infection[a,b]	1.34 (1.30 – 1.37)	<0.001
Stage 2/3 AKI[c]	0.93 (0.85 – 1.03)	0.164
Stage 3 AKI[c]	0.97 (0.87 – 1.08)	0.538

Legend: Controlled for: age, race, sex, number of healthcare encounters, Elixhauser comorbidity score, dialysis utilization, intensive care admissions, and hospital location.

e. Outcomes were not observed at all hospitals; between 1-2% of the population could not be analyzed (MRSA: 20,092 patients, 220,303 encounters; CDiff: 20,092 patients, 219,621 encounters; VRE: 20,092 patients, 219,494 encounters)
f. Any resistant infection defined as pooled occurrence of MRSA, CDiff, or VRE
g. Only patient encounters with at least one SCr analyzed (19,046 patients; 145206 encounters)

eTable 8: Results of Generalized Estimating Equations Sensitivity Analysis Including Length of Stay

Outcome	Odds Ratio (95% CI)	p-value
All-cause mortality	1.02 (0.96 – 1.09)	0.512
MRSA[a]	1.44 (1.36 – 1.52)	<0.001
CDiff[a]	1.04 (0.93 – 1.15)	0.513
VRE[a]	1.17 (1.04 – 1.32)	0.007
Any resistant infection[a,b]	1.32 (1.29 – 1.36)	<0.001
Stage 2/3 AKI	0.93 (0.84 – 1.02)	0.105
Stage 3 AKI	0.94 (0.85 – 1.05)	0.303

Legend: Controlled for: age, race, sex, baseline serum creatinine, number of healthcare encounters, Elixhauser comorbidity score, dialysis utilization, intensive care admissions, hospital location, and length of stay (constrained to a minimum of 1 day).

c. Outcomes were not observed at all hospitals; between 1-2% of the population could not be analyzed (MRSA: 20,092 patients, 220,303 encounters; CDiff: 20,092 patients, 219,621 encounters; VRE: 20,092 patients, 219,494 encounters)
d. Any resistant infection defined as pooled occurrence of MRSA, CDiff, or VRE
e. Only patient encounters with at least one SCr analyzed (19,046 patients; 145206 encounters)

© 2024 Gray MP et al. *JAMA Network Open*.
