# Supplementary material for: Long-Term Outcomes Associated With β-Lactam Allergies
Source: JAMA Netw Open. 2024 May 17;7(5):e2412313. doi: 10.1001/jamanetworkopen.2024.12313 (PMC11102016; doi:10.1001/jamanetworkopen.2024.12313)
Supplement: Supplement 2. — Data Sharing Statement [file jamanetwopen-e2412313-s002.pdf]

Data Sharing Statement

Gray. Long-Term Outcomes Associated With β-Lactam Allergies. *JAMA Netw Open*. Published May 17, 2024. doi:10.1001/jamanetworkopen.2024.12313

Data
Data available: No
